# Modulation of the Immune Response to Allergies Using Alternative Functional Foods

**DOI:** 10.3390/ijms25010467

**Published:** 2023-12-29

**Authors:** Soledad López-Enríquez, Ana M. Múnera-Rodríguez, Camila Leiva-Castro, Francisco Sobrino, Francisca Palomares

**Affiliations:** 1 Department of Medical Biochemistry and Molecular Biology, and Immunology, School of Medicine, University of Seville, Avenue Sanchez Pizjuan s/n, 41009 Seville, Spain; anamunrod2@alum.us.es (A.M.M.-R.); camiferleiva@gmail.com (C.L.-C.); fsobrino@us.es (F.S.); 2 Institute of Biomedicine of Seville (IBiS), Virgen del Rocío University Hospital, Virgen Macarena University Hospital, University of Seville, CSIC, 41013 Seville, Spain

**Keywords:** allergic immune response, functional foods, immune modulation, rhinitis allergic, food allergy and atopic dermatitis

## Abstract

Modulation of the allergic immune response through alternative therapies is a field of study that aims to address allergic reactions differently from traditional approaches. These therapies encompass the utilization of natural functional foods, which have been observed to exert an influence on the immune response, thus mitigating the severity of allergies. Indeed, some studies suggest that the incorporation of these nutraceuticals can regulate immune function, leading to a reduction in histamine release and subsequent alleviation of allergic symptoms. Moreover, certain herbs and dietary supplements, such as curcumin, are believed to possess anti-inflammatory properties, which may serve to moderate allergic responses. Although the results remain somewhat mixed and require further research, these alternative therapies exhibit the potential to impact the allergic immune response, thereby providing complementary options to conventional treatments. Therefore, in this review, we aim to provide an updated account of functional foods capable of modulating the immune response to allergies. In that sense, the review delves into functional foods sourced from plants (phytochemicals), animals, and marine algae. Emphasis is placed on their potential application in the treatment of allergic disorders. It also provides an overview of how these foods can be effectively utilized as functional foods. Additionally, it explores the molecular mechanisms and scientific validity of various bioactive natural compounds in the management of allergies.

## 1. Introduction

The majority of healthy individuals do not exhibit reactions to common and typically innocuous environmental substances. However, an increasing proportion of the population responds abnormally to one or more of these substances [1,2], resulting in the development of allergic reactions with an immunological basis. In such cases, this abnormal response occurs because the immune system is incapable of distinguishing between harmless and pathological substances. These hypersensitivity reactions to innocuous molecules give rise to tissue damage in allergic individuals due to multiple and complex inflammatory processes. Allergic diseases encompass a range of conditions triggered by the immune system’s hypersensitivity to various environmental allergens.

Within allergies, we can determine different types such as food allergy (FA), allergic asthma (AA), atopic dermatitis (AD), allergic rhinitis (AR), conjunctivitis, chronic rhinosinusitis with or without nasal polyposis (CRS or CRSwNP), drug allergy (DA), and venom allergy [3,4,5]. Allergic reactions can manifest in a variety of ways, ranging from mild discomfort to severe and life-threatening responses [6]. Moreover, the prevalence of allergies and allergic skin reactions has been on the rise [7].

These allergic reactions typically involve a sensitization and memory phase as well as an effector phase [8]. Briefly, the key cells in the allergic response include B and T lymphocytes, basophils, eosinophils, and mast cells. Upon first exposure to the allergen, T cells, specifically T helper type 2 (Th2) cells, become activated and release cytokines, such as interleukin (IL)-4 and IL-13. These cytokines stimulate B lymphocytes to generate specific immunoglobulin E (IgE) antibodies against that particular allergen [9]. Thus, in this proinflammatory microenvironment, it has been demonstrated that functional foods can act upon the immune system by modulating or balancing the exacerbated inflammatory response [10].

Nutraceuticals, known for providing health benefits and influencing disease prevention and treatment, can also modulate epigenetic changes [11,12]. Due to this, these compounds act as cellular and functional modulators, contributing to the homeostasis of physiological processes [11]. These compounds have been identified as having health-promoting properties and the potential for therapeutic or preventive applications. In the context of regulating allergic responses, some nutraceuticals can play a significant role due to their anti-inflammatory, antioxidant, and immune system-modulating properties. This review aims to elucidate the anti-inflammatory and anti-allergic effects of nutraceuticals on hypersensitivity reactions, paying special attention to effects on allergic reactions (AR, FA, and AD) and the underlying mechanisms involved in these processes.

## 2. Allergic Diseases

Allergies are complex systemic diseases arising from defects in the immune system [13]. As we have previously described, these pathologies encompass FA, DA, AR, conjunctivitis, CRS or CRSwNP, AA, and AD [6]. The prevalence of allergic diseases, once diagnosed, continues to escalate significantly over time, affecting between 10 and 30% of the global population [2]. Although most allergies may not be reversed, treatments can help to alleviate allergy symptoms. These treatments include antihistamines, nasal steroids, leukotriene receptor antagonists, bronchodilators, topical calcineurin inhibitors (TCI), and immunotherapy, such as monoclonal antibodies and vaccines [14].

Hypersensitivity reactions were classified into I-IV pathophysiologic types by the Gell and Coombs classification [15], re-classified by Rajan [16], and revised by Johansson and collaborators [17]. This classification includes immediate or immunoglobulin (Ig) E-mediated (Type I), antibody-mediated cytotoxic reactions (Type II), immune complex-mediated reactions (Type III), and delayed-type hypersensitivity (Type IV) [18].

### Immunological Mechanism of Allergic Reactions

The immune system responds to antigens, inducing strong immune responses. Both genetic and environmental factors can influence the development of allergic reactions in individuals. Thus, the principal immune cells and molecular mechanisms involved in immune-mediated hypersensitivity reactions [8,14] are as follows: (a) Type I reactions primarily involve mast cells, basophils, and eosinophils, which possess high-affinity FcRI receptors that bind to allergen-specific IgE secreted by plasmatic cells. This binding leads to degranulation and the release of histamine and other inflammatory mediators, such as vasoactive amines and lipid mediators, among others. (b) Type II reactions are characterized by IgG and IgM production by plasma cells, targeting cell surface or extracellular matrix self-antigens. Thus, through opsonization, these antibodies activate the complement system and select cells for phagocytosis by neutrophils and macrophages, ultimately recruiting inflammatory cells and inducing tissue damage. (c) Type III reactions are mediated by IgM and IgG antibodies specific to blood-soluble antigens. The recruitment of immune complexes is facilitated by the complement system and Fc receptor and leukocyte activation. Subsequently, immune complexes are deposited into tissue’s vascular endothelium to cause inflammation, thrombosis, and tissue damage. (d) Type IV reactions represent delayed responses mediated by T cells after antigen-professional cells, such as dendritic cells (DCs), present antigens. Tissue injury may result from CD4+ T lymphocytes, which secrete cytokines inducing inflammation and macrophage activation, or CD8+ cytotoxic T cells, which kill target cells while producing inflammatory cytokines.

To further categorize and achieve a better definition of the complex diversity of these reactions, Lerch and Pichler proposed a subclassification of these type IV reactions into four groups: Type IVa, in which cytotoxicity is produced preferentially by the recruitment and activation of monocytes, such as FA. However, in Type IVb, eosinophils are responsible for cytotoxicity, such as DA, whereas CD4+ and CD8+ T lymphocytes recruit and actively induce cytotoxicity in Type IVc, such as autoimmune diseases. Finally, marked by the activation and preferential recruitment of neutrophils attracted by the chemokine CXCL-8 and the granulocyte-monocyte colony-stimulating factor (GM-CSF), T lymphocytes characterize toxicity in Type IVd, such as coeliac disease [19].

## 3. Functional Foods (Nutraceuticals)

Nutraceuticals play a crucial role in the context of allergies, given their potential to modulate the immune response and reduce inflammation associated with allergic reactions. Derived from various sources, including foods, herbs, specific nutrients, or natural products, these compounds possess properties that can impact the body’s physiology and, in some cases, mitigate allergic symptoms while downregulating mast cell degranulation [10,20]. Some nutraceuticals exhibit anti-inflammatory and antioxidant effects, which can be advantageous in the management of allergies. For instance, certain herbs, such as curcumin, contain compounds that can help mitigate inflammation associated with allergic responses.

This review aims to provide an in-depth exploration of the immunological mechanisms of key nutraceuticals known for their modulatory effects on allergic reactions.

### Therapeutic Nutraceuticals and Their Immunomodulatory Mechanisms

**Figure 1:**
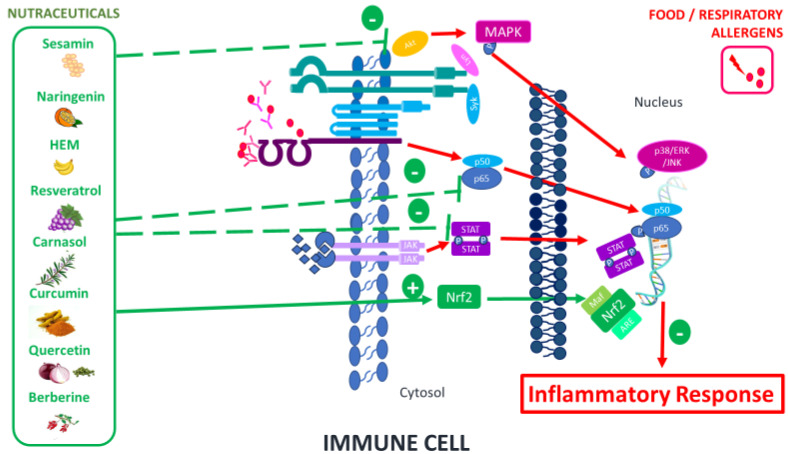
Main signaling pathways modulated by nutraceuticals.

Nutraceuticals have demonstrated their effectiveness in regulating the heightened inflammatory response characteristic of allergic reactions. This approach involves intervention in the signaling pathways activated in this type of response, either using nutraceuticals independently or in combination with conventional medications. This strategy presents an attractive opportunity to induce significant alterations in allergy-related immunocellular properties (Table 1). The signaling pathways modulated by nutraceuticals primarily encompass nuclear factor erythroid 2-related factor 2 (Nrf2), mitogen-activated protein kinases (MAPK), and nuclear factor kappa-light-chain-enhancer of activated B cells (NFκB) (Figure 1).

Sesamin, a natural polyphenolic compound with potent antioxidant effects, has been demonstrated to significantly reduce inflammation in the ovalbumin (OVA)-induced murine model. This reduction is accompanied by a decrease in the expression of cytokines typically associated with a Th2 response pattern, such as IL-4, IL-5, IL-13, and serum IgE levels. In addition, the number of total inflammatory cells and eosinophils decreased in animals treated with sesamin. Furthermore, it was able to reverse the activation of the NFκB signaling pathway [21].

Another study demonstrated that naringenin, a flavanone compound from citrus fruits, significantly reduced OVA-induced airway inflammation and airway reactivity in an animal model. This study showed that, after administering naringenin to these mice, the levels of Th2 cytokines (IL-4 and IL-13) in the bronchoalveolar lavage fluid (BALF) and the total IgE levels in the blood decreased. Naringenin was observed to inhibit the NFκB signaling pathway at the lung level [22]. Similarly, another study demonstrated that naringenin exhibited antioxidant and anti-inflammatory effects in rats with OVA-induced asthma. They described that treatment with this nutraceutical significantly decreased malondialdehyde (MDA), but on the contrary, it highly increased glutathione (GSH) levels and notably reduced the IL-4 and IL-13 levels in lung tissue. Furthermore, the total eosinophil count was significantly reduced [23].

The oral administration of Cyanidin-3-*O*-β-glucoside (C3G), a metabolite produced by *Saccharomyces cerevisiae*, in IgE-sensitized mice before antigen inoculation suppressed passive cutaneous anaphylaxis reaction. The mechanism involved in this suppression was the inhibition of β-hexosaminidase (*β*-Hex) and the release of histamines mediated by IgE, thus preventing mast cell degranulation. These results suggest the potential of C3G for the prevention or treatment of type I allergies [24].

Musa paradisiaca inflorescence (MPI), a banana product with immune bioactivity, has demonstrated immunomodulatory capabilities in a mouse model of combined AR and AS. MPI inhibits type 2 immune cells through the NFκB pathway and decreases the expression levels of the CD86 and HLA-DR markers on human M1 macrophages independent of M2 modulation [25].

Recently, resveratrol, a natural phenol from different plants, has been shown to inhibit mast cell degranulation by decreasing *β*-Hex release through the inhibition of phosphatidylinositol (PI) 4-kinase type II in animal models [10]. Moreover, resveratrol inhibits STAT3 and MAPK (ERK1/2) pathways in human mast cells activated by IgE [10]. Furthermore, resveratrol has also shown, at low concentrations, to exert anti-inflammatory properties through the arachidonic acid pathway, inhibiting prostaglandin D2 (PGD2) biosynthesis and enhancing the release of TNF from human mature mast cells after IgE-dependent activation [26]. In this line, resveratrol decreased the release of β-Hex and histamine in rat basophilic leukemia-2H3 cells, decreasing serum blood-specific IgE. Moreover, resveratrol inhibited the release of β-hex and histamine in bone marrow-derived cells and alleviated mast cell-mediated passive cutaneous anaphylaxis reactions [27]. Another study indicated that oral administration of resveratrol inhibited the immune response in mice with OVA-induced asthma. They demonstrated that the mechanism of action was mediated by an increase in FOXP3+ cells, and subsequently alteration of the miRNAs profile, specifically, resveratrol induced a regulation of miR-34a in lung infiltrating cells [28].

Carnosol, a diterpenoid found in rosemary extracts, exhibits anti-inflammatory and antioxidant effects. Its impact was investigated in an asthma murine model, revealing that carnosol treatment effectively inhibited t mast cell degranulation and decreased eosinophils in the BALF of the asthma murine model. Moreover, carnosol also inhibited inflammatory responses by decreasing IL-4 and IL-13 in both BALF and lungs [29]. In association with this, the main polyphenolic constituent of rosemary extract, carnosic acid (CA), demonstrated the ability to mitigate the allergic response in early and late phases of bone marrow-derived mast cells (BMMCs) sensitized with IgE and stimulated with the allergen. The results showed an inhibition of Syk phosphorylation and a reduction in Akt phosphorylation, along with the blockade of the NFκB pathway. Additionally, CA blocked intracellular Ca^2+^ mobilization, allergen-induced reactive oxygen species (ROS) production, and subsequent mast cell degranulation. Furthermore, CA decreased the release of proinflammatory cytokines and chemokines (IL-6, TNF, IL-13, CCL1, CCL2, CCL3, and CCL9) by selectively inhibiting the activity and phosphorylation of Syk, Akt, and the NFκB pathway in animal models of allergic inflammation [30].

Curcumin, the active compound in the curry spice turmeric, possesses anti-inflammatory properties. In fact, it has been demonstrated that ingestion of curcumin during exposure to oral allergens inhibits mast cell activation and intestinal anaphylaxis in OVA-allergic mice. In this case, what was blocked was the signaling pathway that inhibits phosphorylation of the p56 subunit of NFkB [31].

Piperine, the major alkaloid present in long pepper and black pepper, demonstrates anti-allergic effects. This nutraceutical has been shown to inhibit eosinophil infiltration and the Th2 response in an OVA-induced asthma model [32]. In addition, oral administration of pepper nigrum extract (PNE) in an OVA-induced AR mouse model inhibited early phase allergic nasal symptoms. PNE also blocked the infiltration of inflammatory cells, such as eosinophils, in both nasal lavage fluid (NLF) and nasal tissue. Furthermore, PNE prevented the activation of STAT3 and NFκB signaling pathways, leading to an increased synthesis of Th1 cytokines and the suppression of Th2 and Th17 cytokines [33].

Quercetin, a common polyphenol found in various natural sources like onions and shallots, is an aglycone bioflavonoid. This nutraceutical inhibits the release of allergic mediators (IgE-mediated), such as histamine, TNF-α, IL-1β, IL-6, and IL-8, in rats and human cell lines, such as RBL-2H3 and HMC-1 mast cells, and it also inhibits Ca^2+^ entry and the NFκB signaling pathway [34]. Quercetin has been proposed as a natural treatment for AR [35].

Baicalin is a flavonoid compound present in plants of the genus Scutellaria (Lamiaceae), specifically in leaves and stem bark. Moreover, this compound exhibits anti-allergic and anti-inflammatory activities. Baicalin inhibits the release of histamine, β-hex expression, and the JAK2-STAT5 and NF-kB signaling pathways in human HMC-1 mast cells stimulated with lipopolysaccharide (LPS), blocking the inflammatory response [36]. Furthermore, this nutraceutical has been employed to regulate the immune response in a mouse model of FA [37].

Berberine, a quinoline alkaloid found in plants, possesses anti-inflammatory properties. Berberine has been shown to reduce allergic inflammation in a house dust mite AR mouse model, decreasing levels of specific IgE and transcriptional factors such as GATA-3 and T-bet. Furthermore, this nutraceutical has been shown to increase Treg cells [38]. Additionally, it reduces the release of β-hex, histamine, IL-4, and TNF-α in RBL-2H3 cells and blocks the activation of the MAPK pathway in mouse models of passive cutaneous anaphylaxis [39].

Omega-6 and omega-3 fatty acids, along with their metabolites, have demonstrated positive effects in AS models and allergic diseases [40]. Among them, Fat-1 was employed as a mouse model with increased n-3 or n-6 polyunsaturated fatty acids (PUFA) in tissue. They demonstrated that transgenic Fat-1 mice with OVA-induced allergic airway reaction modulated the Th2-cell response with a decrease in IL-5, IL-9, and IL-13 in BALF [41]. In addition, alpha-linolenic acid (ALA) has been found to decrease AR in an animal model by increasing the production of 15-hydroxyeicosapentaenoic acid (15-HEPE), a metabolite of eicosapentaenoic acid (EPA). In fact, the intranasal injection of 15-HEPE inhibited mast cell degranulation in this animal model [42].

Short-chain fatty acids (SCFAs), such as butyrate and propionate, obtained through microbial fermentation of dietary intake fibers, have demonstrated protective effects against allergic reactions [43,44]. It has been shown that butyrate decreases the activation of human eosinophils. Additionally, this nutraceutical has improved allergen-induced eosinophilia in the pulmonary system and reduced the production of Th2 cytokines (IL-4 and IL-13) in BALF from an OVA-induced pulmonary inflammation mouse model [45].

Dietary fiber, resistant starch (RS), positively influences biomarkers such as glucose metabolism and blood [46,47]. Additionally, RS supplementation can enhance satiety, and its prebiotic effects contribute to a positive impact on the gut microbiome, promoting increased microbial diversity [48]. In vitro studies suggest that fibers like inulin and arabinoxylan hydrolysates can influence the secretion of cytokines and chemokines by epithelial cells, macrophages, and DCs. Fibers can also directly affect immune cells through the activation of pattern recognition receptors (PRRs), including C-type lectin receptors (CLRs), galectins, and Toll-like receptors (TLR-2 and TLR-4). Some fibers, like pectin, may inhibit PRR activation, as demonstrated by its blocking of TLR-2-induced cytokine secretion [49].

Astaxanthin (ASX) is a xanthophyll carotenoid found in various sources, including algae, fungi, plants, seafood, and certain bird species like flamingos. This nutraceutical possesses anti-inflammatory and antioxidant properties [11]. ASX decreases the inflammatory response while reducing the imbalance of Th1/Th2 cells and modulating the production of IL-4, IL-5, and IFN-γ in the BALF in a mouse OVA-induced asthma model [50].

Sulforaphane (SFN), an isothiocyanate of vegetable origin with potent antioxidant and anti-inflammatory properties, regulates the immune response (including the modulation of DCs activity with the specific proliferative response of Treg cells) [51]. Preliminary results from our group determined that SNF inhibits the inflammatory reaction induced by LPS in innate cells through MAPKs (p38, p44, and p42) along with the NKkB signaling pathways. In fact, SFN administration in patients with asthma has shown an improvement in bronchoprotection response, accompanied by increased expression levels of NADH-quinone oxidoreductase 1, indicating the involvement of Nrf2-mediated gene pathways [52]. SFN administration has also reduced neutrophil-induced airway inflammation, inhibited myeloperoxidase, and suppressed Th17 proinflammatory responses. The signaling pathway involved was Nrf2. However, the combination of corticosteroids and SFN has inhibited both innate (neutrophilic and eosinophilic) and adaptive Th17/Th2 responses in the airways, decreasing inflammatory cytokines IL-6, IL-23, and IL-17A during treatment in a mouse model of asthma [53]. Finally, SFN has reversed chronic inflammation by activating the Nrf2 antioxidant signaling pathway, leading to reduced pulmonary mRNA expression of TNFα, SMAD2, IL6, IL-1β, IL-8, and MIP-1β in a chronic allergic disease model induced by OVA in mice [54].

In the following section, the review will discuss the most relevant nutraceutical remedies used in managing patients with AR, FA, and AD.

**Table 1:** Immunomodulatory effect of nutraceuticals in vitro and in vivo models.

Nutraceuticals	Study Design	Immunomodulatory Effect	Refs.
Sesamin	Asthma murine model	↓ NFκB and ↓ (IL-4, IL-5, and IL-13) ↓ IgE	[21]
Naringenin	Asthma/Inflammatory murine model	↓ NFκB and ↓ (IL-4 and IL-13)	[22]
		↓ MDA, ↓ (IL-4 and IL-13) in lung tissues, and ↓ total eosinophils ↑ GSH	[23]
C3G	Cutaneous reaction in murine model	↓ *β*-Hex and ↓ histamine release IgE-mediated. ↓ Mast cell degranulation	[24]
MPI	Murine model, combining AR and asthma and human M1 macrophages	↑ Th1 profile ↓ Type 2 immune cells ↓ NFκB pathway ↓ CD86 and ↓ HLA-DR	[25]
Resveratrol	Murine model	↓ Mast cells degranulation with ↓ *β*-Hex by ↓ PI 4-kinase ↓ IgE-mediated histamine release	[10]
	Human mast cells from AR patients	↓ IgE- mediated *β*-Hex release ↓ pSTAT3 and pERK1/2 ↓ Nasal symptoms in AR patients with ↓ (IgE, IL-4, and TNF-*α*) ↓ Eosinophils in blood	
	Human mast cells from AR patients	↓ PGD2 ↑ TNF in mast cells after IgE-dependent activation	[26]
	Rat basophilic leukemia-2H3 cells and AR murine model	↓ *β*-Hex ↓ IgE-mediated histamine ↓ (DCs and B and mast cells) ↓ TXNIP pathway ↓ (PGD2, LTC4, ECP, IL-4, IL-5, IL-6, IL-33, and TNF)	[27]
	Asthma murine model	↑ FOXP3+ cells ↓ miR-34a in lung infiltrating cells	[28]
Carnosol	Asthma murine model	↓ Mast cells degranulation ↓ Eosinophils ↓ (IL-4 and IL-13)	[29]
	Allergic inflammation model	↓ (Syk, Akt phosphorylation, and NFκB). ↓ Intracellular Ca^2+^ mobilization ↓ ROS production and β-Hex release ↓ Mast cells degranulation, and ↓ (IL-6, TNF, IL-13, CCL1, CCL2, CCL3, and CCL9)	[30]
Curcumin	Allergic inflammation model	↓ Mast cell activation and ↓ NFκB signaling pathway	[31]
Piperine	Asthma and AR murine model	↓ Eosinophil infiltration ↓ Th2 response and ↑ Th1 cells ↓ STAT3 and NFκB signaling pathways	[32,33]
Quercetin	Human HMC-1 mast cells from AR patients	↓ (Histamine, TNF-α, IL-1β, IL-6, and IL-8) and Ca^2+^ ↓ NFκB signaling pathway ↓ Allergic symptoms in AR patients	[34]
Baicalein	AR and FA murine model	↓ (Histamine, OVA-IgE, IL-1β, IL-6, IL-8, and TNF) ↑ Treg in a mouse	[36]
	Human HMC-1 mast cells	↓ (Histamine and β-hex) ↓ JAK2-STAT5 and NF-kB signaling pathways	[37]
Berberine	House dust mite AR murine model	↓ (sIgE, GATA-3, and T-bet mRNA levels, L-10) ↓ Eosinophil infiltration and ↑Treg	[38]
	Cutaneous anaphylaxis murine model	↓ Mast cell activation by ↓ (β-hex, histamine, IL-4 and TNF) ↓ MAPK signaling pathway	[39]
PUFAs	Transgenic Fat-1 murine model (allergic airway)	↓ Th2-cell response	[41]
	AR animal model	↓ degranulation mast cells	[42]
SCFAs	Keratinocyte humans	Protection and reprogramming of skin barrier function and metabolism	[43]
	Pulmonary inflammation murine model	↓ Eosinophils ↓ Th2 (IL-4 and IL13)	[45]
Fiber (RS)	In vitro model: HEK-Blue™ TLR cells and macrophage cells RAW264.7	↑ Modulation of immune cells and chemokine secretion via CLRs or TLRs	[49]
ASX	Asthma murine model	↓ Th1/Th2 cells ↓ (IL-4 and IL-5) and ↑ IFN-γ) and ↓ IgE	[50]
SFN	Asthma patients	↑ Bronchoprotective response ↓ Nrf2 signaling pathway	[52]
	Asthma animal model	↑ antioxidant effects ↓ Th17 responses	[53]
		mRNA ↓ (TNF-α, SMAD2, IL6, IL-1β, IL-8, and MIP-1β) expression	[54]

Abbreviations: AR, allergic rhinitis; MDA: malondialdehyde; *β*-Hex: β-hexosaminidase; PI 4-kinase: phosphatidylinositol 4-kinases; PGD2: prostaglandin D2; TXNIP: thioredoxin-interacting protein; SYK: Tyrosine-protein kinase SYK; ROS: Reactive oxygen species; PUFAs: polyunsaturated fatty acids; SCFA: Short-chain fatty acids; SFN: Sulphoraphane; ASX: Astaxanthin; C3G: Cyanidin-3-*O*-β-glucoside; MPI: Musa paradisiaca inflorescence; RS: Resistant starch; CLRs: C-type lectin receptors; TLRs: Toll-like receptors. ↓ (reduction). ↑ (increase).

## 4. Nutraceuticals and Allergic Rhinitis (AR)

AR is a chronic inflammatory respiratory disorder associated with IgE [55]. The immunological response in AR involves a variety of inflammatory cells, including mast cells, T cells, B cells, macrophages, and eosinophils. Recent research has identified immune cells such as ILC2s, Th2 cells, DCs, and epithelial cells as important players in AR pathogenesis [56]. Exposure to allergens activates DCs, initiating a cascade that involves Th2 cells, cytokine release, production of allergen-specific IgE by B cells, and the triggering of allergic responses through mast cells and basophils [57,58].

There are different treatments for AR that control the exaggerated immune response to allergens and alleviate symptoms, such as antihistamines, nasal corticosteroids, antileukotrienes (montelukast), and specific allergen immunotherapy (AIT) [56,59]. However, these treatment approaches may prove ineffective in certain instances. Consequently, both in vitro and in vivo research have been conducted to create AR models, and these investigations also evaluate the impact of various nutraceuticals (Table 2).

Quercetin and SFN have been investigated for their ability to reduce the allergic response by inhibiting the release of inflammatory mediators, thereby helping to relieve the symptoms of the pathology [60,61]. One study examined the impact of quercetin on the production of nitric oxide (NO) in human nasal epithelial cells (HNEpC) following IL-4 stimulation. The findings of the study suggested that quercetin may have a therapeutic effect on AR by influencing the behaviour of HNEpC cells [62]. Thioredoxin (TRX) is a protein known for regulating oxidative metabolism and neutralizing reactive oxygen species, which play a role in suppressing allergic inflammation [63]. A study determined quercetin’s impact on AR symptoms and TRX production in nasal cells both in vitro and in vivo. Results showed that taking quercetin orally notably decreased nasal symptoms and raised TRX levels in nasal fluids. This boost in TRX production by quercetin might enhance its effectiveness in treating AR [64]. Recently, it has been investigated the anti-allergic effects of the oral intake of quercetin as a supplement on allergen-induced reactions in AR adult subjects in a randomized controlled trial. Their results showed that allergic symptoms improved after 4 weeks of repeated oral administrations [65]. In a clinical study conducted with a randomized, double-blind design and a placebo control group, SFN demonstrated its effectiveness in reducing type 2 cytokines such as IL-4, IL-5, and IL-13 found in the mucus of the nasal cavity in AR patients compared to the control group. Furthermore, nasal symptoms (TNSS) and peak nasal inspiratory flow (PNIF) showed notable improvements after 3 weeks of treatment with SFN [66].

Subsequently, in a clinical trial featuring a double-blind, randomized, and placebo-controlled design, AR patients who received resveratrol exhibited a reduction in nasal symptoms, serum IgE, IL-4, TNF-α, and eosinophil levels, as compared to the AR patients in the placebo group [28]. In fact, resveratrol also presented anti-allergic and anti-inflammatory effects by inhibiting the thioredoxin-interacting protein (TXNIP), decreasing specific IgE levels, and reducing inflammatory mediators in an AR model [27].

In an animal model of OVA-induced AR, the potential therapeutic benefits of a piper nigrum extract were explored. The mice that were treated with piper nigrum extract presented a notable reduction in histamine release from mast cells, a decrease in nasal symptoms, and a reduction in eosinophil infiltration. Furthermore, the extract played a protective role in preventing nasal epithelial barrier dysfunction by elevating the expression of the active form of Nrf2, thereby promoting the synthesis of the anti-inflammatory enzyme HO-1 [33].

Similar results were found for baicalin, which showed an inhibitory effect on allergic response in OVA-induced AR pigs and in LPS-stimulated human mast cells [36]. Berberine reduced allergic inflammation in a house dust mite AR mouse model, decreasing the specific-IgE levels and increasing the regulatory response [38].

Omega-3 fatty acids (EPA) are found in fatty fish and have anti-inflammatory properties that could help reduce inflammation in AR [67]. In an in vivo study using an animal model of AR, it was found that linseed oil, abundant in ALA, lessens AR by encouraging eosinophils to produce 15-HEPE, an EPA metabolite. Notably, 15-HEPE, generated by eosinophils, suppressed allergic symptoms by hindering mast cell degranulation through peroxisome proliferator-activated receptor gamma. This suggests a promising treatment for AR [42].

In the study of dietary fiber, a single-blinded randomized controlled trial investigated the impact of a 15 mg dose of dried Ma-al-Shaheer (a traditional Iranian medicine based on barley, Hordeum vulgare) compared to a twice-daily dosage of 60 mg fexofenadine (an antihistamine) in AR patients over a 21-day period. Both groups exhibited improved AR control, and symptoms were significantly reduced in both sets of participants. However, the reduction in nasal congestion, post-nasal drip, and headache appeared slightly more pronounced in the Ma-al-Shaheer group [68].

**Table 2:** Achievements of nutraceuticals as alternative therapies in AR.

Nutraceuticals	Refs.	Source of Nutraceuticals	Phase of Allergic Reaction	Achievements	Limitations
Quercetin	[62,63,64,65]	Isolated from a natural source. Commercially obtained	In vitro models under specific IL-14 stimulation. Onset phase in animal model, after sensitization phase. Clinical trial in AR patients in the sensitization phase.	Suppressive effect on NO production from nasal epithelial cells. Increase of TRX production in nasal epithelial cells and animal models. Significant decrease in allergy nasal symptoms (seizing and rubbing).	Lack of food matrix Low bioavailability Lack of research on dosage and forms of administration Limited research in humans and clinical trials Lack of studies of possible interactions with other drugs
Sulforaphane	[66]	Broccoli sprout extract, including sulphoraphane.	Clinical trial in AR patients in the sensitization phase.	Anti-inflammatory and anti-allergic properties in AR patients, with the improvement of nasal symptoms.	
Resveratrol	[27,28]	Isolated from natural sources (for example, *Abies georgei*).	Onset phase in animal model, after sensitization phase.	Reduction of nasal symptoms. Anti-allergic and anti-inflammatory properties.	
Piper	[69]	Piper nigrum extract.		Reduction of nasal symptoms and inflammatory mediators.	
Baicalin	[36]	Isolated from natural sources. Commercially obtained.		Anti-allergic response in OVA-induced AR pigs and in LPS-stimulated human mast cells.	
Berberine	[38]	Isolated from natural sources. Commercially obtained.		Reduction of the inflammatory and increase in regulatory response.	
Omega-3 fatty acids	[67]	Dietary fatty acids on allergic models.	Before the sensitization phase in animal models.	Suppression of the allergic symptoms.	
Fiber	[68]	Natural formulation from *Hordeum vulgare.*	Clinical trial in AR patients in the sensitization phase.	All symptoms of AR except cough were significantly reduced.	

Abbreviations: AR, allergic rhinitis; TRX, thioredoxin; NO: Nitric oxide; OVA: *Ovalbumin*; LPS: Lipopolysaccharide.

## 5. Nutraceuticals and Food Allergy (FA)

FA results from abnormal immune responses to food antigens, primarily leaning towards Th2 responses linked with IL-4, IL-5, and IL-13. Current treatments for IgE-mediated FA mainly focus on avoiding suspected allergens and utilizing antihistamines and corticosteroid therapies, which exhibit low efficacy and several side effects. Immunotherapy for food allergens aims to desensitize and establish lasting immune tolerance by gradually increasing exposure to these allergens [70]. However, this process has a high incidence of adverse reactions and requires long-term treatment [9,70]. Therefore, there is a need to develop new alternative therapies, such as nutraceuticals (Table 3).

In the last few years, nutraceuticals have gained attention for their potential in preventing and treating food allergies (FA). A study utilized in vitro models, including human basophil KU812 cell degranulation and Caco-2 monolayer cells, to investigate the impact of various nutraceuticals (baicalein, luteolin, isorhamnetin, and naringenin) on the allergenicity of ω-5 gliadin peptides and their association with damage to Caco-2 intestinal epithelial monolayers. The findings indicate that these nutraceuticals effectively inhibit KU812 cell degranulation induced by ω-5 gliadin-derived peptides, decrease the release of IL-6 and TNF-α, and enhance intestinal barrier function. These results suggest the potential pharmaceutical use of these nutraceuticals in treating FA [69].

Furthermore, the effects of C3G in an OVA-induced FA mouse were described. The results of this study determined that C3G ameliorated clinical FA symptoms and regulated Th1/Th2 immune balance in the intestinal mucosa (an increase in IFN-γ and a decrease in IL-4 and TNF-α) [71].

Another study indicated that resveratrol may have the potential to alleviate food hypersensitivity or allergic diseases. The results showed that the nutraceutical was not only able to suppress the development of allergic symptoms and decrease the serum level of specific IgE but also decreased the population of DCs, B cells, and mast cells in OVA-induced allergic mice [27].

In addition, bisdemethoxycurcumin (BDMC), an important ingredient derived from curcumin, has been reported as a treatment for mast cell-mediated food allergic diseases. The results showed that BDMC was able to regulate the Th1/Th2 immune balance and inhibit the activation of MAPK and NFκB pathways in FA animal models [72]. Moreover, protein disulfide isomerases regulated IgE-mediated mast cell responses, and their inhibition with curcumin and quercetin conferred protective effects during FA [73]. Regarding quercetin, it has been shown that the conjugation of this nutraceutical with iron could offer protection against allergic reactions to milk (specifically beta-lactoglobulin) [74].

Piper nigrum (PN) is widely employed for its antioxidant, anti-allergic, anti-tumor, anti-inflammatory, anti-diarrheal, and gastrointestinal protective properties. To explore its potential, an OVA-induced FA mouse model with varying concentrations of PN extract was utilized. The outcomes revealed that PN extract mitigated FA symptoms, reduced IgE levels, and upregulated Treg cell-associated cytokines [75].

Moreover, some new formulas derived from herbal formula 2 for food allergy have been designed, such as EBF-2. From this formula, a powerful active compound, berberine, has been identified. This nutraceutical is a potent IgE suppressor, associated with the cellular regulation of immunometabolism in IgE-producing plasma cells and postulated to be a potent therapeutic tool for IgE-mediated FA in animal models for peanut allergy [76]. In fact, berberine combined with oral immunotherapy to peanuts has also been shown to induce tolerance, which is sustained for a long time and is associated with a specific microbiome in the FA murine model concerning peanuts [77].

Omega-3 polyunsaturated fatty acids are believed to have protective effects on human health by influencing immune responses, as has been observed in FA [78]. Explained by PUFAs, they have shown that in an in vivo FA mouse model, they modulated the activity of basophils in the sensitization phase of allergy and subsequent Th2 cytokine release [79,80]. Regarding this, supplementation in the diet with omega-3 fatty acids during pregnancy has influenced the development of FA in offspring and has played a role in the immune system as an anti-inflammatory agent, providing cell membrane stabilization with inhibition of antigen presentation in FA [81,82,83]. Indeed, a study has shown that a diet rich in these PUFAs helps reduce adverse effects in children with fish allergies [84].

Metabolites produced by gut bacteria, such as SCFAs, have a significant impact on both immune cells and the gut. Recent data suggest that some dietary components, such as SCFAs, may promote intestinal homeostasis and suppress FA [85]. Recent research findings are intriguing, as they indicate that SCFA butyrate demonstrates a direct impact on mast cells by epigenetically controlling the signaling molecules associated with FcεRI [86]. Importantly, elevated levels of the SCFAs butyrate and propionate found in feces during early childhood are linked to a decreased risk of FA [87]. Novel findings highlight an essential role in supporting epithelial barrier integrity, oral tolerance, and protection against FA through the involvement of dietary fiber and SCFAs. This is supported by observations that children diagnosed with a cow’s milk allergy exhibited lower levels of fecal butyrate compared to healthy controls [88].

**Table 3:** Achievements of nutraceuticals as alternative therapies in FA.

Nutraceuticals	Refs.	Source of Nutraceuticals	Phase of Allergic Reaction	Achievements	Limitations
Naringenin and Baicalein	[69]	Isolated from a natural source. Commercially obtained.	In vitro models.	Reduction of basophils degranulation and improvement of the intestinal epithelial barrier.	Lack of research on dosage and forms of administration. Limited research in humans and clinical trials. Variability in the results obtained in the models studied. They do not replace food allergen avoidance.
C3G	[71]	Commercially obtained.	Onset phase in animal model, after sensitization phase.	Improve the clinical FA symptoms and regulate the T cell phenotype.	
Resveratrol	[27]	Isolated from natural sources (for example, *Abies georgei*).		Attenuation of allergic responses in anaphylactic models.	
BDMC	[72]	Commercially obtained.		Attenuation of OVA-induced FA inhibiting the MAPK and NF-κB signaling pathways.	
Quercetin	[73,74]	Commercially obtained and specific conjugation.	In vitro model. Before the sensitization phase in animal model.	Regulation of IgE-mediated mast cell responses and protection in front of effects during FA. Iron–quercetin complex confers resilience in cow’s milk allergy.	
Pipper	[75]	Piper nigrum extract.	Onset phase in animal model, after sensitization phase.	FA attenuation, decreasing Th2 cell response, and regulating the Th17/Treg balance.	
Berberine	[76,77]	Commercially obtained, and specific conjugation.	Onset phase in animal model, after sensitization phase.	Combination with OIT induces tolerance to peanuts.	
PUFAs	[80,81,83,84]	Omega-3 supplementation.	FA patients in the sensitization phase.	Prevention of FA in children and reduction in adverse effects in fish allergy.	
Fiber and SCFAs	[85,86,88]	Modification to the AIN93G control diet. Commercially obtained (SCFAs).	Onset phase in animal model, after sensitization phase. Children with non-IgE-mediated CMA.	Induction of the tolerance and protection in FA. Regulation mast cells degranulation (via epigenetic). Regulation of non-IgE mediated CMA in children.	

Abbreviations: C3G: Cyanidin-3-O-β-glucoside; FA: Food allergy; BDMC: Bisdemethoxycurcumin; OVA: *Ovalbumin*; MAPK: mitogen-activated protein kinases; NF-κB: nuclear factor kappa B; IgE: immunoglobulin E; OIT: oral immunotherapy; PUFAs: polyunsaturated fatty acids; SCFAs: short-chain fatty acids; CMA: cow’s milk allergy.

## 6. Nutraceuticals and Atopic Dermatitis (AD)

Atopic dermatitis (AD), commonly known as eczema, is a chronic inflammatory skin condition characterized by red, itchy, and inflamed skin. AD typically emerges in early childhood but can affect individuals of any age. While the exact cause of AD remains incompletely understood, it is believed to result from a combination of genetic, environmental, and immune system factors. The condition’s severity can vary, with symptoms ranging from mild, occasional itching to severe and persistent rashes. Management typically involves a multifaceted approach, including the avoidance of triggers, skin moisturizing, the use of topical treatments (like corticosteroids or calcineurin inhibitors), and, in some cases, oral medications may be necessary. However, some nutraceuticals have been explored for their possible effects on symptom management.

In this context, it has been shown that the use of naringenin protects mice against AD (induced by dinitrochlorobenzene) by inhibiting inflammation through the JAK2/STAT3 pathway and promoting skin wound healing [89,90].

Furthermore, in an animal model of passive cutaneous anaphylaxis (PCA), C3G was used and results indicated its capacity to suppress the PCA response at a low dose. Therefore, it was concluded that this nutraceutical could serve as a new treatment for AD [24]. Other studies related to resveratrol showed an attenuation of AD, postulating it as a therapeutic agent [91,92]. The results of these studies showed a reduction in AD symptoms, inflammation, oxidative damage, and inflammatory cytokines in the in vivo AD model [93]. Recently, nanoparticles combined with resveratrol, containing linolenic acid or hyaluronic acid hydrogel, have been designed as new tools for AD treatment [94,95].

On the other hand, carnosol from *Rosmarinus officinalis* L. extract has also been suggested as a treatment for AD flares [96]. In a mouse model of AD induced by phthalic anhydride, carnosol was found to reduce skin inflammation and inhibit the expression of nitric oxide synthase (iNOS) and cyclooxygenase (COX-2) in skin tissue [97]. This effect coincided with the suppression of STAT3 activation in skin tissue [98].

Other studies reported that curcumin is a potent regulator of skin disorders [99]. In fact, the nutraceutical has been shown to ameliorate OVA-induced skin pathology in animal models by suppressing inflammatory cell infiltration in the dermal region and by reducing STAT6 phosphorylation and GATA3 expression [100]. Just as with other nutraceuticals, the design of new therapeutic approaches, such as nanoparticles or microneedles, is also being carried out in combination with curcumin to achieve an effective, rapid, and sustained delivery system for the treatment of AD [101].

Several experiments have revealed that quercetin presents beneficial effects on AD [102]. In vitro and in vivo, quercetin has been determined to enhance inflammatory cytokines, as well as markedly suppress Nf-kB and phosphorylation of Erk1/2 and JNK DNCB induced in an AD animal model [103]. In addition, it suppresses IgE production and mast cell infiltration and improves wounds in the skin of animals affected by AD [104].

Another study yielded similar results for the nutraceutical baicalin, which improved skin lesions in atopic dermatitis induced by DFNB by modulating the intestinal microbiota and the JAK/STAT pathway [105]. In addition, berberine induced anti-AD effects by downregulating cutaneous eukaryotic translation initiation factor 3 subunit F and mucosa-associated lymphoid tissue lymphoma translocation protein 1 in mice with AD [106].

Numerous studies have explored the use of PUFAs in various common skin diseases [107]. The supplementation with PUFAs, such as docosahexaenoyl ethanolamide, mitigated cutaneous inflammation and regulated the synthesis and activity of cytokines by promoting wound healing in animal and clinical models [108]. SCFAs exhibit anti-inflammatory effects, as described in the previous section. Free fatty acid receptor 2 (FFA2, formerly known as GPR43) is a specific receptor for SCFAs, such as acetate, which regulates the inflammatory response. In this sense, an FFA2 agonist has shown to be a potent candidate as a therapeutic agent for AD induced by DNCB. Mice treated with this agonist reduced IgE levels, skin hypertrophy, and mast cell accumulation, suggesting that FFA2 could be a therapeutic target for AD [109]. In addition, *Lactococcus chungangensis* CAU 28 (CAU 28) cream cheese was tested in an AD BALB/c mouse. The results found that it led to the upregulation of SCFA levels, modulation in the gut microbiota, and a reduction in AD symptoms [110]. These findings align with similar reports concerning the modulation of the microbiota and the reduction in inflammation in AD [111,112,113].

In a retrospective cross-sectional case–control study focusing on fiber, it was observed that Japanese adults with AD and poor antihistamine control did not show a significant association between total fiber intake and urticaria control test scores [114].

Studies have demonstrated that both a combination of extract of *Centella asiatica* and astaxanthin (ASX) has anti-inflammatory effects in a phthalic anhydride (PA) mouse model of AD. This combination inhibited the expression of iNOS and COX-2, NFκB activity, and the release of TNF-α, IL-6 and IgE, compared to those compounds administered alone [115,116]. Similar results have been found with sulforaphane, which led to a reduction in symptoms in AD, inhibiting oxidative stress, DNA oxidation, inflammation, and apoptosis [117]. Moreover, it demonstrated a therapeutic effect in an AD murine model by activating the Nrf2/HO-1 axis [118].

## 7. Conclusions

Nutraceuticals are bioactive substances found in foods or are concentrated extracts that possess beneficial health properties, demonstrating both preventive and therapeutic effects in certain conditions. In the case of allergies, their potential to modulate the immune system and reduce allergic responses has been a focal point of investigation, as we presented in this review.

We can conclude that some nutraceuticals, including quercetin, SFN, PUFAs, SCFAs, and dietary fibers, have demonstrated a certain ability to modulate the immune response. They influence the regulation of the immune system, which could impact the manifestation and severity of allergic reactions while reducing inflammation. Some of these nutraceuticals are currently undergoing clinical trials, contributing to advancing research on the efficacy and safety of using nutraceuticals as anti-allergic treatments in patients. The objective is to determine whether they are more effective than current treatments or have fewer side effects.

While certain nutraceuticals, like curcumin or ASX, have the potential to reduce allergic symptoms, addressing specific limitations requires further research. In this sense, more studies are required to delve into the effectiveness of these bioactive foods and the need for clinical trials to determine whether they can be used as standalone therapies or as complementary approaches to established treatments. Furthermore, most studies are focused on the use of these nutraceuticals in a purified form, which presents certain disadvantages, such as the lack of studies involving their complete use within their food matrix or the potential for side effects. In fact, a high concentration of purified nutraceuticals could increase the risk of side effects and using them in a purified form could lead to the loss of complex interactions with the food matrix, influencing bioavailability and absorption.

Despite these limitations, no adverse effects were identified in recent studies examining the use of bioactive foods. However, it is essential to address recommendations for future studies concerning PUFAs, SCFAs, and dietary fibers. Currently, there is a notable absence of research on the impact of PUFAs, SCFAs, and dietary fibers during pregnancy, as existing studies primarily focus on fiber interventions. Exploring this early opportunity for allergy prevention through additional intervention trials is crucial. Additionally, careful consideration is required regarding the dosage of specific fiber types, especially regarding the potential for adverse metabolic effects. Understanding the cause-and-effect relationship between fiber intake, microbiota metabolism, and immune system dysfunction is imperative. Furthermore, exploring metabolites generated by microbial fermentation of dietary fibers, such as SCFAs, as potential novel therapeutic agents or immunotherapy adjuvants warrants investigation.

In summary, although there is research suggesting the potential of nutraceuticals to help control the progression of allergic responses, it is essential to advance research to corroborate their potent anti-allergic role.

## Data Availability

Not applicable.

## Abbreviations

AA	Allergic asthma
AD	Atopic dermatitis
AR	Allergic rhinitis
ASX	Astaxanthin
ALA	Alpha-linolenic acid
AIT	Allergen immunotherapy
BALF	Bronchoalveolar lavage fluid
β-Hex	β-hexosaminidase
BMMCs	Bone marrow-derived mast cells
BDMC	Bisdemethoxycurcumin
CRS	Chronic rhinosinusitis
CRSwNP	Chronic rhinosinusitis without nasal polyposis
C3G	Cyanidin-3-O-β-glucoside
CA	Carnosic acid
COX-2	Cycloocxygenase
DA	Drug allergy
DCs	Dendritic cells
EPA	Eicosapentaenoic acid
FA	Food allergy
GM-CSF	Granulocyte- monocyte colony-stimulating factor.
GPR43- FFA2	Free fatty acid receptor 2
TXNIP	Thioredoxin-interacting protein.
GSH	Highly increased glutation
HNEpC	Human nasal epithelial cells
iNOS	Nitric oxide synthase
IL	Interleukin
Ig	Immunoglobulin
IgE	Inmunoglobulin E
LPS	Lipopolysaccharide
MAPK	Mitogen-activated protein kinases
MDA	Malondialdehyde
MPI	Musa paradisiaca inflorescence
NO	Nitric oxide.
Nrf2	Nuclear factor Erythroid 2- related factor 2
NFκB	Kappa-light-chain-enhancer
NLF	Nasal lavage fluid
PA	Phthalic anhydride
PI	Phosphatidylinositol
PGD	Prostaglandin D2
PNE	Piper nigrum extract
PCA	Passive cutaneous anaphylaxis
PUFA	Polyunsaturated fatty acids
PNIF	Peak nasal inspiratory flow
OVA	Ovalbumin
ROS	Allergen-induced reactive oxygen species
RS	Resistant starch
SCFAs	Short-chain fatty acids
SFN	Sulforaphane
TCI	Topical calcineurin inhibitors
TRX	Thioredoxin
Th2	T helper type 2 cells
TNSS	aNsal symptoms
15-HEPE	15-hydroxyeicosapentaenoic acid

## References

[1] Abbas A.K., Lichtman A.H., Pillai S. (2022). Cellular and Molecular Immunology.

[2] Shin Y.H., Hwang J., Kwon R., Lee S.W., Kim M.S., Shin J.I., Yon D.K., GBD 2019 Allergic Disorders Collaborators (2023). Global, regional, and national burden of allergic disorders and their risk factors in 204 countries and territories, from 1990 to 2019: A systematic analysis for the Global Burden of Disease Study 2019. Allergy.

[3] Samitas K., Carter A., Kariyawasam H.H., Xanthou G. (2018). Upper and lower airway remodelling mechanisms in asthma, allergic rhinitis and chronic rhinosinusitis: The one airway concept revisited. Allergy.

[4] Fokkens W.J., Lund V.J., Hopkins C., Hellings P.W., Kern R., Reitsma S., Toppila-Salmi S., Bernal-Sprekelsen M., Mullol J., Alobid I. (2020). European Position Paper on Rhinosinusitis and Nasal Polyps 2020. Rhinology.

[5] Golden D.B. (2015). Anaphylaxis to insect stings. Immunol. Allergy Clin. N. Am..

[6] Breiteneder H., Peng Y.Q., Agache I., Diamant Z., Eiwegger T., Fokkens W.J., Traidl-Hoffmann C., Nadeau K., O’Hehir R.E., O’Mahony L. (2020). Biomarkers for diagnosis and prediction of therapy responses in allergic diseases and asthma. Allergy.

[7] Pacheco S.E., Guidos-Fogelbach G., Annesi-Maesano I., Pawankar R., Amato G.D., Latour-Staffeld P., Urrutia-Pereira M., Kesic M.J., Hernandez M.L. (2021). Climate change and global issues in allergy and immunology. J. Allergy Clin. Immunol..

[8] Palomares O., Akdis M., Martin-Fontecha M., Akdis C.A. (2017). Mechanisms of immune regulation in allergic diseases: The role of regulatory T and B cells. Immunol. Rev..

[9] Brasal-Prieto M., Fernandez-Prades L., Dakhaoui H., Sobrino F., Lopez-Enriquez S., Palomares F. (2023). Update on In Vitro Diagnostic Tools and Treatments for Food Allergies. Nutrients.

[10] Civelek M., Bilotta S., Lorentz A. (2022). Resveratrol Attenuates Mast Cell Mediated Allergic Reactions: Potential for Use as a Nutraceutical in Allergic Diseases?. Mol. Nutr. Food Res..

[11] Alba G., Dakhaoui H., Santa-Maria C., Palomares F., Cejudo-Guillen M., Geniz I., Sobrino F., Montserrat-de la Paz S., Lopez-Enriquez S. (2023). Nutraceuticals as Potential Therapeutic Modulators in Immunometabolism. Nutrients.

[12] Vranceanu M., Galimberti D., Banc R., Dragos O., Cozma-Petrut A., Heghes S.C., Vostinaru O., Cuciureanu M., Stroia C.M., Miere D. (2022). The Anticancer Potential of Plant-Derived Nutraceuticals via the Modulation of Gene Expression. Plants.

[13] Galli S.J., Tsai M., Piliponsky A.M. (2008). The development of allergic inflammation. Nature.

[14] Basketter D.A., McFadden J.P., Kimber I. (2012). Assessing the severity of allergic reactions: A regulatory dilemma. Contact Dermat..

[15] Wang J., Zhou Y., Zhang H., Hu L., Liu J., Wang L., Wang T., Zhang H., Cong L., Wang Q. (2023). Pathogenesis of allergic diseases and implications for therapeutic interventions. Signal Transduct. Target. Ther..

[16] Rajan T.V. (2003). The Gell-Coombs classification of hypersensitivity reactions: A re-interpretation. Trends Immunol..

[17] Johansson S.G., Bieber T., Dahl R., Friedmann P.S., Lanier B.Q., Lockey R.F., Motala C., Ortega Martell J.A., Platts-Mills T.A., Ring J. (2004). Revised nomenclature for allergy for global use: Report of the Nomenclature Review Committee of the World Allergy Organization, October 2003. J. Allergy Clin. Immunol..

[18] Dispenza M.C. (2019). Classification of hypersensitivity reactions. Allergy Asthma Proc..

[19] Lerch M., Pichler W.J. (2004). The immunological and clinical spectrum of delayed drug-induced exanthems. Curr. Opin. Allergy Clin. Immunol..

[20] McCarty M.F., Lerner A., DiNicolantonio J.J., Benzvi C. (2021). Nutraceutical Aid for Allergies-Strategies for Down-Regulating Mast Cell Degranulation. J. Asthma Allergy.

[21] Lin C.H., Shen M.L., Zhou N., Lee C.C., Kao S.T., Wu D.C. (2014). Protective effects of the polyphenol sesamin on allergen-induced T(H)2 responses and airway inflammation in mice. PLoS ONE.

[22] Shi Y., Dai J., Liu H., Li R.R., Sun P.L., Du Q., Pang L.L., Chen Z., Yin K.S. (2009). Naringenin inhibits allergen-induced airway inflammation and airway responsiveness and inhibits NF-kappaB activity in a murine model of asthma. Can. J. Physiol. Pharmacol..

[23] Jasemi S.V., Khazaei H., Fakhri S., Mohammadi-Noori E., Farzaei M.H. (2022). Naringenin Improves Ovalbumin-Induced Allergic Asthma in Rats through Antioxidant and Anti-Inflammatory Effects. Evid. Based Complement. Altern. Med..

[24] Hiemori-Kondo M., Morikawa E., Fujikura M., Nagayasu A., Maekawa Y. (2021). Inhibitory effects of cyanidin-3-O-glucoside in black soybean hull extract on RBL-2H3 cells degranulation and passive cutaneous anaphylaxis reaction in mice. Int. Immunopharmacol..

[25] Gadelha F.A.A.F., Cavalcanti R.F.P., Vieira G.C., Ferreira L.K.D.P., de Sousa G.R., Filho J.M.B., Barbosa M.A., dos Santos S.G., Piuvezam M.R. (2021). Immunomodulatory properties of Musa paradisiaca L. inflorescence in Combined Allergic Rhinitis and Asthma Syndrome (CARAS) model towards NFκB pathway inhibition. J. Funct. Foods.

[26] Shirley D., McHale C., Gomez G. (2016). Resveratrol preferentially inhibits IgE-dependent PGD2 biosynthesis but enhances TNF production from human skin mast cells. Biochim. Biophys. Acta.

[27] Zhang Y.F., Liu Q.M., Gao Y.Y., Liu B., Liu H., Cao M.J., Yang X.W., Liu G.M. (2019). Attenuation of allergic responses following treatment with resveratrol in anaphylactic models and IgE-mediated mast cells. Food Funct..

[28] Alharris E., Alghetaa H., Seth R., Chatterjee S., Singh N.P., Nagarkatti M., Nagarkatti P. (2018). Resveratrol Attenuates Allergic Asthma and Associated Inflammation in the Lungs Through Regulation of miRNA-34a That Targets FoxP3 in Mice. Front. Immunol..

[29] Lee J.E., Im D.S. (2021). Suppressive Effect of Carnosol on Ovalbumin-Induced Allergic Asthma. Biomol. Ther..

[30] Crozier R.W.E., Yousef M., Coish J.M., Fajardo V.A., Tsiani E., MacNeil A.J. (2023). Carnosic acid inhibits secretion of allergic inflammatory mediators in IgE-activated mast cells via direct regulation of Syk activation. J. Biol. Chem..

[31] Kinney S.R., Carlson L., Ser-Dolansky J., Thompson C., Shah S., Gambrah A., Xing W., Schneider S.S., Mathias C.B. (2015). Curcumin Ingestion Inhibits Mastocytosis and Suppresses Intestinal Anaphylaxis in a Murine Model of Food Allergy. PLoS ONE.

[32] Kim S.H., Lee Y.C. (2009). Piperine inhibits eosinophil infiltration and airway hyperresponsiveness by suppressing T cell activity and Th2 cytokine production in the ovalbumin-induced asthma model. J. Pharm. Pharmacol..

[33] Bui T.T., Fan Y., Piao C.H., Nguyen T.V., Shin D.U., Jung S.Y., Hyeon E., Song C.H., Lee S.Y., Shin H.S. (2020). Piper Nigrum extract improves OVA-induced nasal epithelial barrier dysfunction via activating Nrf2/HO-1 signaling. Cell. Immunol..

[34] Park H.H., Lee S., Son H.Y., Park S.B., Kim M.S., Choi E.J., Singh T.S., Ha J.H., Lee M.G., Kim J.E. (2008). Flavonoids inhibit histamine release and expression of proinflammatory cytokines in mast cells. Arch. Pharm. Res..

[35] Thornhill S.M., Kelly A.M. (2000). Natural treatment of perennial allergic rhinitis. Altern. Med. Rev..

[36] Zhou Y.J., Wang H., Sui H.H., Li L., Zhou C.L., Huang J.J. (2016). Inhibitory effect of baicalin on allergic response in ovalbumin-induced allergic rhinitis guinea pigs and lipopolysaccharide-stimulated human mast cells. Inflamm. Res..

[37] Bae M.J., Shin H.S., See H.J., Jung S.Y., Kwon D.A., Shon D.H. (2016). Baicalein induces CD4(+)Foxp3(+) T cells and enhances intestinal barrier function in a mouse model of food allergy. Sci. Rep..

[38] Kim B.Y., Park H.R., Jeong H.G., Kim S.W. (2015). Berberine reduce allergic inflammation in a house dust mite allergic rhinitis mouse model. Rhinology.

[39] Fu S., Ni S., Wang D., Fu M., Hong T. (2019). Berberine suppresses mast cell-mediated allergic responses via regulating FcvarepsilonRI-mediated and MAPK signaling. Int. Immunopharmacol..

[40] Miyata J., Arita M. (2015). Role of omega-3 fatty acids and their metabolites in asthma and allergic diseases. Allergol. Int..

[41] Bilal S., Haworth O., Wu L., Weylandt K.H., Levy B.D., Kang J.X. (2011). Fat-1 transgenic mice with elevated omega-3 fatty acids are protected from allergic airway responses. Biochim. Biophys. Acta.

[42] Sawane K., Nagatake T., Hosomi K., Hirata S.I., Adachi J., Abe Y., Isoyama J., Suzuki H., Matsunaga A., Fukumitsu S. (2019). Dietary Omega-3 Fatty Acid Dampens Allergic Rhinitis via Eosinophilic Production of the Anti-Allergic Lipid Mediator 15-Hydroxyeicosapentaenoic Acid in Mice. Nutrients.

[43] Trompette A., Pernot J., Perdijk O., Alqahtani R.A.A., Domingo J.S., Camacho-Munoz D., Wong N.C., Kendall A.C., Wiederkehr A., Nicod L.P. (2022). Gut-derived short-chain fatty acids modulate skin barrier integrity by promoting keratinocyte metabolism and differentiation. Mucosal Immunol..

[44] Chen C., Liu C., Zhang K., Xue W. (2023). The role of gut microbiota and its metabolites short-chain fatty acids in food allergy. Food Sci. Hum. Wellness.

[45] Theiler A., Barnthaler T., Platzer W., Richtig G., Peinhaupt M., Rittchen S., Kargl J., Ulven T., Marsh L.M., Marsche G. (2019). Butyrate ameliorates allergic airway inflammation by limiting eosinophil trafficking and survival. J. Allergy Clin. Immunol..

[46] Sobh M., Montroy J., Daham Z., Sibbald S., Lalu M., Stintzi A., Mack D., Fergusson D.A. (2022). Tolerability and SCFA production after resistant starch supplementation in humans: A systematic review of randomized controlled studies. Am. J. Clin. Nutr..

[47] Wang Y., Chen J., Song Y.H., Zhao R., Xia L., Chen Y., Cui Y.P., Rao Z.Y., Zhou Y., Zhuang W. (2019). Effects of the resistant starch on glucose, insulin, insulin resistance, and lipid parameters in overweight or obese adults: A systematic review and meta-analysis. Nutr. Diabetes.

[48] Venter C., Meyer R.W., Greenhawt M., Pali-Scholl I., Nwaru B., Roduit C., Untersmayr E., Adel-Patient K., Agache I., Agostoni C. (2022). Role of dietary fiber in promoting immune health-An EAACI position paper. Allergy.

[49] Sahasrabudhe N.M., Beukema M., Tian L., Troost B., Scholte J., Bruininx E., Bruggeman G., van den Berg M., Scheurink A., Schols H.A. (2018). Dietary Fiber Pectin Directly Blocks Toll-Like Receptor 2-1 and Prevents Doxorubicin-Induced Ileitis. Front. Immunol..

[50] Hwang Y.H., Hong S.G., Mun S.K., Kim S.J., Lee S.J., Kim J.J., Kang K.Y., Yee S.T. (2017). The Protective Effects of Astaxanthin on the OVA-Induced Asthma Mice Model. Molecules.

[51] Fernandez-Prades L., Brasal-Prieto M., Alba G., Martin V., Montserrat-de la Paz S., Cejudo-Guillen M., Santa-Maria C., Dakhaoui H., Granados B., Sobrino F. (2023). Sulforaphane Reduces the Chronic Inflammatory Immune Response of Human Dendritic Cells. Nutrients.

[52] Brown R.H., Reynolds C., Brooker A., Talalay P., Fahey J.W. (2015). Sulforaphane improves the bronchoprotective response in asthmatics through Nrf2-mediated gene pathways. Respir. Res..

[53] Al-Harbi N.O., Nadeem A., Ahmad S.F., AlThagfan S.S., Alqinyah M., Alqahtani F., Ibrahim K.E., Al-Harbi M.M. (2019). Sulforaphane treatment reverses corticosteroid resistance in a mixed granulocytic mouse model of asthma by upregulation of antioxidants and attenuation of Th17 immune responses in the airways. Eur. J. Pharmacol..

[54] Royce S.G., Licciardi P.V., Beh R.C., Bourke J.E., Donovan C., Hung A., Khurana I., Liang J.J., Maxwell S., Mazarakis N. (2022). Sulforaphane prevents and reverses allergic airways disease in mice via anti-inflammatory, antioxidant, and epigenetic mechanisms. Cell. Mol. Life Sci..

[55] Savoure M., Bousquet J., Jaakkola J.J.K., Jaakkola M.S., Jacquemin B., Nadif R. (2022). Worldwide prevalence of rhinitis in adults: A review of definitions and temporal evolution. Clin. Transl. Allergy.

[56] Zhang Y., Lan F., Zhang L. (2022). Update on pathomechanisms and treatments in allergic rhinitis. Allergy.

[57] Nur Husna S.M., Tan H.T., Md Shukri N., Mohd Ashari N.S., Wong K.K. (2022). Allergic Rhinitis: A Clinical and Pathophysiological Overview. Front. Med..

[58] Kabashima K., Nakashima C., Nonomura Y., Otsuka A., Cardamone C., Parente R., De Feo G., Triggiani M. (2018). Biomarkers for evaluation of mast cell and basophil activation. Immunol. Rev..

[59] Lam H.Y., Tergaonkar V., Ahn K.S. (2020). Mechanisms of allergen-specific immunotherapy for allergic rhinitis and food allergies. Biosci. Rep..

[60] Jafarinia M., Sadat Hosseini M., Kasiri N., Fazel N., Fathi F., Ganjalikhani Hakemi M., Eskandari N. (2020). Quercetin with the potential effect on allergic diseases. Allergy Asthma Clin. Immunol..

[61] Ashifha S., Vijayashree J., Vudayana K., Chintada D., Unnikrishnan P. (2023). A Study of Cutaneous Adverse Drug Reactions at a Tertiary Care Center in Andhra Pradesh, India. Cureus.

[62] Ebihara N., Takahashi K., Takemura H., Akanuma Y., Asano K., Sunagawa M. (2018). Suppressive Effect of Quercetin on Nitric Oxide Production from Nasal Epithelial Cells In Vitro. Evid. Based Complement. Alternat. Med..

[63] Wang J., Zhou J., Wang C., Fukunaga A., Li S., Yodoi J., Tian H. (2022). Thioredoxin-1: A Promising Target for the Treatment of Allergic Diseases. Front. Immunol..

[64] Edo Y., Otaki A., Asano K. (2018). Quercetin Enhances the Thioredoxin Production of Nasal Epithelial Cells In Vitro and In Vivo. Medicines.

[65] Yamada S., Shirai M., Inaba Y., Takara T. (2022). Effects of repeated oral intake of a quercetin-containing supplement on allergic reaction: A randomized, placebo-controlled, double-blind parallel-group study. Eur. Rev. Med. Pharmacol. Sci..

[66] Yusin J., Wang V., Henning S.M., Yang J., Tseng C.H., Thames G., Arnold I., Heber D., Lee R.P., Sanavio L. (2021). The Effect of Broccoli Sprout Extract on Seasonal Grass Pollen-Induced Allergic Rhinitis. Nutrients.

[67] Venter C., Meyer R.W., Nwaru B.I., Roduit C., Untersmayr E., Adel-Patient K., Agache I., Agostoni C., Akdis C.A., Bischoff S.C. (2019). EAACI position paper: Influence of dietary fatty acids on asthma, food allergy, and atopic dermatitis. Allergy.

[68] Derakhshan A., Khodadoost M., Ghanei M., Gachkar L., Hajimahdipour H., Taghipour A., Yousefi J., Khoshkhui M., Azad F.J. (2019). Effects of a Novel Barley-Based Formulation on Allergic Rhinitis: A Randomized Controlled Trial. Endocr. Metab. Immune Disord. Drug Targets.

[69] Wu S., Zhang R., Liu Y., Gao J., Wu Y., Tu C., Chen H., Yuan J. (2022). In Vitro Effect of Flavonoids on Basophils Degranulation and Intestinal Epithelial Barrier Damage Induced by omega-5 Gliadin-Derived Peptide. Foods.

[70] Mayorga C., Palomares F., Canas J.A., Perez-Sanchez N., Nunez R., Torres M.J., Gomez F. (2021). New Insights in Therapy for Food Allergy. Foods.

[71] Li J., Zou C., Liu Y. (2022). Amelioration of Ovalbumin-Induced Food Allergy in Mice by Targeted Rectal and Colonic Delivery of Cyanidin-3-O-Glucoside. Foods.

[72] Wang Y., Zhang P., Zhang J., Hong T. (2022). Bisdemethoxycurcumin attenuates OVA-induced food allergy by inhibiting the MAPK and NF-kappaB signaling pathways. Exp. Ther. Med..

[73] Krajewski D., Polukort S.H., Gelzinis J., Rovatti J., Kaczenski E., Galinski C., Pantos M., Shah N.N., Schneider S.S., Kennedy D.R. (2020). Protein Disulfide Isomerases Regulate IgE-Mediated Mast Cell Responses and Their Inhibition Confers Protective Effects During Food Allergy. Front. Immunol..

[74] Roth-Walter F., Afify S.M., Pacios L.F., Blokhuis B.R., Redegeld F., Regner A., Petje L.M., Fiocchi A., Untersmayr E., Dvorak Z. (2021). Cow’s milk protein beta-lactoglobulin confers resilience against allergy by targeting complexed iron into immune cells. J. Allergy Clin. Immunol..

[75] Wang L., Jia X., Yu Q., Shen S., Gao Y., Lin X., Zhang W. (2021). Piper nigrum extract attenuates food allergy by decreasing Th2 cell response and regulating the Th17/Treg balance. Phytother Res..

[76] Yang N., Maskey A.R., Srivastava K., Kim M., Wang Z., Musa I., Shi Y., Gong Y., Fidan O., Wang J. (2023). Inhibition of pathologic immunoglobulin E in food allergy by EBF-2 and active compound berberine associated with immunometabolism regulation. Front. Immunol..

[77] Srivastava K., Cao M., Fidan O., Shi Y., Yang N., Nowak-Wegrzyn A., Miao M., Zhan J., Sampson H.A., Li X.M. (2023). Berberine-containing natural-medicine with boiled peanut-OIT induces sustained peanut-tolerance associated with distinct microbiota signature. Front. Immunol..

[78] Hoppenbrouwers T., Cvejic Hogervorst J.H., Garssen J., Wichers H.J., Willemsen L.E.M. (2019). Long Chain Polyunsaturated Fatty Acids (LCPUFAs) in the Prevention of Food Allergy. Front. Immunol..

[79] Hoppenbrouwers T., Fogliano V., Garssen J., Pellegrini N., Willemsen L.E.M., Wichers H.J. (2020). Specific Polyunsaturated Fatty Acids Can Modulate in vitro Human moDC2s and Subsequent Th2 Cytokine Release. Front. Immunol..

[80] Sartorio M.U.A., Pendezza E., Coppola S., Paparo L., D’Auria E., Zuccotti G.V., Berni Canani R. (2021). Potential Role of Omega-3 Polyunsaturated Fatty Acids in Pediatric Food Allergy. Nutrients.

[81] Feketea G., Kostara M., Bumbacea R.S., Vassilopoulou E., Tsabouri S. (2023). Vitamin D and Omega-3 (Fatty Acid) Supplementation in Pregnancy for the Primary Prevention of Food Allergy in Children-Literature Review. Children.

[82] Jerzynska A., Polanska A., Trafalska E., Jankowska A., Podlecka D., Brzozowska A. (2023). Prenatal polyunsaturated fatty acids and atopic dermatitis and food allergy in children from Polish Mother and Child Cohort study. Int. J. Occup. Med. Environ. Health.

[83] Huynh L.B.P., Nguyen N.N., Fan H.Y., Huang S.Y., Huang C.H., Chen Y.C. (2023). Maternal Omega-3 Supplementation During Pregnancy, but Not Childhood Supplementation, Reduces the Risk of Food Allergy Diseases in Offspring. J. Allergy Clin. Immunol. Pract..

[84] Pecoraro L., Dalle Carbonare L., Castagnoli R., Marseglia G.L., Piacentini G., Pietrobelli A. (2022). IgE-mediated fish allergy in children: Is omega-3 supplementation useful?. Int. J. Food Sci. Nutr..

[85] Tan J., McKenzie C., Vuillermin P.J., Goverse G., Vinuesa C.G., Mebius R.E., Macia L., Mackay C.R. (2016). Dietary Fiber and Bacterial SCFA Enhance Oral Tolerance and Protect against Food Allergy through Diverse Cellular Pathways. Cell Rep..

[86] Folkerts J., Redegeld F., Folkerts G., Blokhuis B., van den Berg M.P.M., de Bruijn M.J.W., van Ijcken W.F.J., Junt T., Tam S.Y., Galli S.J. (2020). Butyrate inhibits human mast cell activation via epigenetic regulation of FcepsilonRI-mediated signaling. Allergy.

[87] Roduit C., Frei R., Ferstl R., Loeliger S., Westermann P., Rhyner C., Schiavi E., Barcik W., Rodriguez-Perez N., Wawrzyniak M. (2019). High levels of butyrate and propionate in early life are associated with protection against atopy. Allergy.

[88] Berni Canani R., De Filippis F., Nocerino R., Paparo L., Di Scala C., Cosenza L., Della Gatta G., Calignano A., De Caro C., Laiola M. (2018). Gut microbiota composition and butyrate production in children affected by non-IgE-mediated cow’s milk allergy. Sci. Rep..

[89] Tian L., Wang M., Wang Y., Li W., Yang Y. (2023). Naringenin ameliorates atopic dermatitis by inhibiting inflammation and enhancing immunity through the JAK2/STAT3 pathway. Genes Genom..

[90] Sun R., Liu C., Liu J., Yin S., Song R., Ma J., Cao G., Lu Y., Zhang G., Wu Z. (2023). Integrated network pharmacology and experimental validation to explore the mechanisms underlying naringenin treatment of chronic wounds. Sci. Rep..

[91] Lee C.H., Yang H., Park J.H.Y., Kim J.E., Lee K.W. (2022). Piceatannol, a metabolite of resveratrol, attenuates atopic dermatitis by targeting Janus kinase 1. Phytomedicine.

[92] Shen Y., Xu J. (2019). Resveratrol Exerts Therapeutic Effects on Mice with Atopic Dermatitis. Wounds.

[93] Bangash Y., Saleem A., Akhtar M.F., Anwar F., Akhtar B., Sharif A., Khan M.I., Khan A. (2023). Pterostilbene reduces the progression of atopic dermatitis via modulating inflammatory and oxidative stress biomarkers in mice. Inflammopharmacology.

[94] Cassano R., Serini S., Curcio F., Trombino S., Calviello G. (2022). Preparation and Study of Solid Lipid Nanoparticles Based on Curcumin, Resveratrol and Capsaicin Containing Linolenic Acid. Pharmaceutics.

[95] Conte R., De Luca I., Valentino A., Cerruti P., Pedram P., Cabrera-Barjas G., Moeini A., Calarco A. (2023). Hyaluronic Acid Hydrogel Containing Resveratrol-Loaded Chitosan Nanoparticles as an Adjuvant in Atopic Dermatitis Treatment. J. Funct. Biomater..

[96] Nakagawa S., Hillebrand G.G., Nunez G. (2020). Rosmarinus officinalis L. (Rosemary) Extracts Containing Carnosic Acid and Carnosol are Potent Quorum Sensing Inhibitors of Staphylococcus aureus Virulence. Antibiotics.

[97] Lee D.Y., Hwang C.J., Choi J.Y., Park M.H., Song M.J., Oh K.W., Son D.J., Lee S.H., Han S.B., Hong J.T. (2017). Inhibitory Effect of Carnosol on Phthalic Anhydride-Induced Atopic Dermatitis via Inhibition of STAT3. Biomol. Ther..

[98] Yeo I.J., Park J.H., Jang J.S., Lee D.Y., Park J.E., Choi Y.E., Joo J.H., Song J.K., Jeon H.O., Hong J.T. (2019). Inhibitory effect of Carnosol on UVB-induced inflammation via inhibition of STAT3. Arch. Pharm. Res..

[99] Vollono L., Falconi M., Gaziano R., Iacovelli F., Dika E., Terracciano C., Bianchi L., Campione E. (2019). Potential of Curcumin in Skin Disorders. Nutrients.

[100] Sharma S., Sethi G.S., Naura A.S. (2020). Curcumin Ameliorates Ovalbumin-Induced Atopic Dermatitis and Blocks the Progression of Atopic March in Mice. Inflammation.

[101] Chen Y.L., Chang C.C., Lin Y.C., Chen M.C. (2023). Double-layered PLGA/HA microneedle systems as a long-acting formulation of polyphenols for effective and long-term management of atopic dermatitis. Biomater. Sci..

[102] Rakha A., Umar N., Rabail R., Butt M.S., Kieliszek M., Hassoun A., Aadil R.M. (2022). Anti-inflammatory and anti-allergic potential of dietary flavonoids: A review. Biomed. Pharmacother..

[103] Lee H.N., Shin S.A., Choo G.S., Kim H.J., Park Y.S., Kim B.S., Kim S.K., Cho S.D., Nam J.S., Choi C.S. (2018). Anti-inflammatory effect of quercetin and galangin in LPS-stimulated RAW264.7 macrophages and DNCB-induced atopic dermatitis animal models. Int. J. Mol. Med..

[104] Beken B., Serttas R., Yazicioglu M., Turkekul K., Erdogan S. (2020). Quercetin Improves Inflammation, Oxidative Stress, and Impaired Wound Healing in Atopic Dermatitis Model of Human Keratinocytes. Pediatr. Allergy Immunol. Pulmonol..

[105] Wang L., Xian Y.F., Loo S.K.F., Ip S.P., Yang W., Chan W.Y., Lin Z.X., Wu J.C.Y. (2022). Baicalin ameliorates 2,4-dinitrochlorobenzene-induced atopic dermatitis-like skin lesions in mice through modulating skin barrier function, gut microbiota and JAK/STAT pathway. Bioorg. Chem..

[106] Andoh T., Yoshihisa Y., Rehman M.U., Tabuchi Y., Shimizu T. (2021). Berberine induces anti-atopic dermatitis effects through the downregulation of cutaneous EIF3F and MALT1 in NC/Nga mice with atopy-like dermatitis. Biochem. Pharmacol..

[107] Balic A., Vlasic D., Zuzul K., Marinovic B., Bukvic Mokos Z. (2020). Omega-3 Versus Omega-6 Polyunsaturated Fatty Acids in the Prevention and Treatment of Inflammatory Skin Diseases. Int. J. Mol. Sci..

[108] Huang T.H., Wang P.W., Yang S.C., Chou W.L., Fang J.Y. (2018). Cosmetic and Therapeutic Applications of Fish Oil’s Fatty Acids on the Skin. Mar. Drugs.

[109] Kang J., Im D.S. (2020). FFA2 Activation Ameliorates 2,4-Dinitrochlorobenzene-Induced Atopic Dermatitis in Mice. Biomol. Ther..

[110] Kim J.H., Kim K., Kim W. (2019). Cream Cheese-Derived Lactococcus chungangensis CAU 28 Modulates the Gut Microbiota and Alleviates Atopic Dermatitis in BALB/c Mice. Sci. Rep..

[111] Lee Y.H., Kalailingam P., Delcour J.A., Fogliano V., Thanabalu T. (2023). Olive-Derived Antioxidant Dietary Fiber Modulates Gut Microbiota Composition and Attenuates Atopic Dermatitis Like Inflammation in Mice. Mol. Nutr. Food Res..

[112] Lee M.J., Park Y.M., Kim B., Tae I.H., Kim N.E., Pranata M., Kim T., Won S., Kang N.J., Lee Y.K. (2022). Disordered development of gut microbiome interferes with the establishment of the gut ecosystem during early childhood with atopic dermatitis. Gut Microbes.

[113] Chen P.C., Lo Y.H., Huang S.Y., Liu H.L., Yao Z.K., Chang C.I., Wen Z.H. (2022). The anti-inflammatory properties of ethyl acetate fraction in ethanol extract from Sarcodia suiae sp. alleviates atopic dermatitis-like lesion in mice. Biosci. Biotechnol. Biochem..

[114] Matano Y., Morita T., Ito M., Okazaki S., Koto M., Ichikawa Y., Takayama R., Hoashi T., Saeki H., Kanda N. (2020). Dietary habits in Japanese patients with chronic spontaneous urticaria. Australas. J. Dermatol..

[115] Lee Y.S., Jeon S.H., Ham H.J., Lee H.P., Song M.J., Hong J.T. (2020). Improved Anti-Inflammatory Effects of Liposomal Astaxanthin on a Phthalic Anhydride-Induced Atopic Dermatitis Model. Front. Immunol..

[116] Park J.H., Yeo I.J., Jang J.S., Kim K.C., Park M.H., Lee H.P., Han S.B., Hong J.T. (2019). Combination Effect of Titrated Extract of Centella asiatica and Astaxanthin in a Mouse Model of Phthalic Anhydride-Induced Atopic Dermatitis. Allergy Asthma Immunol. Res..

[117] Alyoussef A. (2022). Attenuation of experimentally induced atopic dermatitis in mice by sulforaphane: Effect on inflammation and apoptosis. Toxicol. Mech. Methods.

[118] Wu W., Peng G., Yang F., Zhang Y., Mu Z., Han X. (2019). Sulforaphane has a therapeutic effect in an atopic dermatitis murine model and activates the Nrf2/HO-1 axis. Mol. Med. Rep..

